# Competition matters: Determining the drivers of land snail community assembly among limestone karst areas in northern Vietnam

**DOI:** 10.1002/ece3.3984

**Published:** 2018-03-26

**Authors:** Parm Viktor von Oheimb, Katharina C. M. von Oheimb, Takahiro Hirano, Tu Van Do, Hao Van Luong, Jonathan Ablett, Sang Van Pham, Fred Naggs

**Affiliations:** ^1^ Life Sciences Department The Natural History Museum London UK; ^2^ Center for Northeast Asian Studies Tohoku University Sendai Japan; ^3^ Department of Aquatic Ecology and Water Environment Institute of Ecology and Biological Resources Vietnam Academy of Science and Technology Ha Noi Vietnam; ^4^ Centre for Rescue and Conservation of Organisms Hoang Lien National Park Sa Pa Vietnam; ^5^ Department of Specimen Preparation and Exhibitive Design Vietnam National Museum of Nature Vietnam Academy of Science and Technology Ha Noi Vietnam

**Keywords:** collection‐based research, Cyclophoridae, *Cyclophorus*, molecular phylogenetics, morphometrics, Southeast Asia

## Abstract

The insular limestone karsts of northern Vietnam harbor a very rich biodiversity. Many taxa are strongly associated with these environments, and individual species communities can differ considerably among karst areas. The exact processes that have shaped the biotic composition of these habitats, however, remain largely unknown. In this study, the role of two major processes for the assembly of snail communities on limestone karsts was investigated, interspecific competition and filtering of taxa due to geographical factors. Communities of operculate land snails of the genus *Cyclophorus* were studied using the dry and fluid‐preserved specimen collections of the Natural History Museum, London. Phylogenetic distances (based on a Bayesian analysis using DNA sequence data) and shell characters (based on 200 semilandmarks) were used as proxies for ecological similarity and were analyzed to reveal patterns of overdispersion (indicating competition) or clustering (indicating filtering) in observed communities compared to random communities. Among the seven studied karst areas, a total of 15 *Cyclophorus* lineages were found. Unique communities were present in each area. The analyses revealed phylogenetic overdispersion in six and morphological overdispersion in four of seven karst areas. The pattern of frequent phylogenetic overdispersion indicated that competition among lineages is the major process shaping the *Cyclophorus* communities studied. The Coastal Area, which was phylogenetically overdispersed, showed a clear morphological clustering, which could have been caused by similar ecological adaptations among taxa in this environment. Only the community in the Cuc Phuong Area showed a pattern of phylogenetic clustering, which was partly caused by an absence of a certain, phylogenetically very distinct group in this region. Filtering due to geographical factors could have been involved here. This study shows how museum collections can be used to examine community assembly and contributes to the understanding of the processes that have shaped karst communities in Vietnam.

## 1 INTRODUCTION

The limestone karst outcrops of Southeast and East Asia, such as those of northern Vietnam, are inhabited by exceptionally large numbers of plant and animal taxa (Clements et al., 2008; Do, 2001; Sterling, Hurley, & Le, 2006). Many biota living in these habitats are strongly associated with the calcium‐rich limestone environment, for example, plant taxa of the families Primulaceae, Begoniaceae, and Orchidaceae as well as species of primates, bats, rodents, and shelled gastropods (Chung et al., 2014; Clements, Sodhi, Schilthuizen, & Ng, 2006; Furey, Mackie, & Racey, 2010; Gao, Ai, Kong, Kang, & Huang, 2015; Musser, Lunde, & Nguyen, 2006; Nadler, Vu, & Streicher, 2007; Páll‐Gergely et al., 2015; Raheem et al., 2017; Zhu, Wang, Li, & Sirirugsa, 2003). For such organisms, karst areas can act as functional islands: The calcareous limestone provides suitable habitats, whereas surrounding regions, which are often characterized by acidic soil, act as dispersal barriers (Clements et al., 2008; Tweedie, 1961). Numerous limestone‐bound organisms are threatened as limestone rocks are being quarried in many Asian countries, including Vietnam, and used as construction material and for the production of concrete (Clements et al., 2006, 2008; Deharveng & Bedos, 2012; Schilthuizen, Liew, Bin Elahan, & Lackman‐Ancrenaz, 2005). Increasing knowledge about limestone biota, their patterns of biodiversity, and underlying processes is therefore of fundamental importance. Vietnam is still relatively poorly studied compared to other regions of high biodiversity due to decades of war and political isolation (Dang, 2008; Schileyko, 2011; Sterling & Hurley, 2005; Sterling et al., 2006; Vermeulen & Maassen, 2003).

Almost a fifth of Vietnam's territory is covered by limestone karst, with most of it being located in the north of the country (Dang, 1996; Do, 2001). The scattered karst areas differ considerably in size and in degree of isolation and are partly distributed along climatic gradients. This complex, island‐like system could have played a major role in shaping and maintaining the very rich total biodiversity of northern Vietnam as individual limestone karst areas harbor unique communities of taxa (a pattern reported from various regions in Southeast Asia; Clements et al., 2006, 2008; Foon, Clements, & Liew, 2017; Sterling et al., 2006; Tongkerd, Lee, Panha, Burch, & Ó Foighil, 2004; Tweedie, 1961). The exact processes that have shaped the biotic composition of these habitats, however, still remain unclear.

Two major processes are believed to be generally involved in the assembly of species communities: (1) competition between taxa and (2) filtering of taxa due to geographical factors (Pausas & Verdú, 2010). If competition is the major process involved, the coexistence of ecologically similar species in the same area is assumed to be restricted by competitive exclusion (Connor & Simberloff, 1979). In contrast, filtering due to geographical factors limits the set of potentially “available” taxa within an area, either due to environmental conditions that prevent the establishment of certain lineages (i.e., habitat filtering), or due to barriers that exclude particular taxa, for example such without specific dispersal abilities (Poff, 1997; Weiher & Keddy, 1995). The two processes result in two generally contrary patterns of community structure. While competition leads to increased ecological dissimilarity among taxa in one community, filtering due to geographical factors leads to increased ecological similarity.

In this study, the influence of these two processes for limestone karst communities in northern Vietnam was investigated using a collection‐based approach. Museum collections represent invaluable archives of biodiversity, which have often been assembled over centuries. The Natural History Museum, London (NHM), harbors one of the world's largest collections of terrestrial gastropods from Vietnam, which includes numerous dry shells as well as fluid‐preserved specimens. Such a collection offers great opportunities to tackle relevant ecological and biogeographical questions as long as certain limitations are acknowledged (see also Pyke & Ehrlich, 2010). In many cases, purely historical collections comprise only one individual per population, are affected by sampling biases (e.g., due to a focus on rare species or deviating individuals), are not suitable for molecular works, and lack adequate documentation (such as geographical data). To overcome these potential caveats, it is essential to incorporate more recent collections that have been systematically sampled, are preserved in a way that allows molecular study (e.g., in ethanol), and have been adequately documented. However, even recent museum collections based on broad‐scale sampling campaigns (in terms of geographical area and taxa) are often not designed to cover certain regions or particular species in detail. For studying community assembly with a collection‐based approach, it is therefore important to focus on well‐sampled areas and prominent model taxa.

The land snail genus *Cyclophorus* Monfort, 1810 (Caenogastropoda: Cyclophoridae), is an example of such a prominent taxon. These large operculate gastropods are among the most conspicuous invertebrates inhabiting the limestone karsts of northern Vietnam. *Cyclophorus* spp. are very abundant, easy to collect, and numerous individuals are present in the NHM's Vietnamese land snail collections. A large part of this material was obtained during a number of field trips to Vietnam in the 2000s and 2010s (some of them conducted as part of a Darwin Initiative project; Naggs, Panha, & Raheem, 2006). During these field trips, it was aimed to systematically collect multiple specimens of all occurring terrestrial gastropod species at each sampling locality. Thus, it can be considered as very unlikely that regularly occurring *Cyclophorus* taxa from well‐covered areas are missing in these collections. Individual samples from these field trips are linked to detailed geographic information and most of them are preserved in ethanol.

A relatively high number of different *Cyclophorus* species have been reported from northern Vietnam (Dang, 2008; Kobelt, 1908), and several taxa can be found on each of the larger karst outcrops (Vermeulen & Maassen, 2003), with individual communities being present in different limestone karst areas (P.V. and K.C.M. von Oheimb, personal observation). Besides limestone karsts, *Cyclophorus* spp. also occur in adjacent nonlimestone habitats, but at much lower abundances (P.V. and K.C.M. von Oheimb, personal observation; see also Tweedie (1961) for the Malay Peninsula). It can thus be assumed that populations of these snails in different karst areas are not completely isolated (as it could also be the case for most other limestone‐associated organisms; Clements et al., 2006; Schilthuizen, Chai, Kimsin, & Vermeulen, 2003; Vermeulen & Maassen, 2003). The ecology of *Cyclophorus* spp. is not well known. The snails are generally detritivorous, mainly ground dwelling, and can be typically found in leaf litter (Kasinathan, Chandramohan, & Natarajan, 1973; Nantarat et al., 2014b; Yen, 1943; P.V. and K.C.M. von Oheimb, personal observation).

Community assembly processes can be examined by studying distribution patterns of ecologically relevant characters of co‐occurring taxa. Among the members of a community, competition is expected to result in a higher dissimilarity of such characters than expected by chance (overdispersion), while filtering due to geographical factors is expected to result in a higher similarity (clustering) (Webb, Ackerly, McPeek, & Donoghue, 2002). The phylogenetic relatedness of taxa is a character that can provide information about similarities in ecological niches. More closely related taxa are assumed to share more phylogenetically conserved traits (Webb et al., 2002), such as physiological characters or life history strategies (for a detailed discussion about this relationship see Losos, 2008 and Cavender‐Bares, Kozak, Fine, & Kembel, 2009). Using phylogenetic relatedness as the only proxy for ecological similarity may have, however, serious pitfalls (discussed by Gerhold, Cahill, Winter, Bartish, & Prinzing, 2015). In phylogenetically distant species of polygyrid land snails, for instance, similar shell shapes and sizes were found where they inhabit the same environment (Emberton, 1995). Convergent evolution of characters can increase the (ecological) similarity of only distantly related taxa. In contrast, different local selection pressures may lead to a relatively fast accumulation of ecological differences within phylogenetic lineages (Gerhold et al., 2015). Closely related camaenid land snail taxa from the Bonin Islands in the Pacific Ocean, for example, have evolved different shell shapes, which correspond to different habitats (Chiba, 2004). Such mechanisms can cause a loss of correlation between phylogenetic relationship and ecology and may remain unnoticed when relying solely on data from molecular phylogenetics.

To overcome these potential obstacles, this study does not only focus on phylogenetic relatedness but also on morphological similarity. As the morphology of an organism is generally associated with its ecology (Ricklefs & Miles, 1994), it partly corresponds to its ecological niche. The most convenient and well‐established morphological character set for most snails is the shell. Similarities in the morphology of gastropod shells are generally known to be associated with similar ecological adaptations (e.g., Chiba, 2004; Goodfriend, 1986; Raheem, Breugelmans, Wade, Naggs, & Backeljau, 2017; Stankowski & Johnson, 2014), while competitive exclusion of taxa with similar shell forms has been reported for several terrestrial gastropod taxa (e.g., Deli & Szekeres, 2011; Uit De Weerd, Groenenberg, Schilthuizen, & Gittenberger, 2006). By including data from both molecular phylogenetics and morphometric shell analyses, mechanisms such as convergent evolution and adaptation to local environments can be recognized (e.g., Emerson, 2002). By making use of test statistics where observed communities are compared to random communities, patterns of overdispersion and clustering can be detected. This allows clarification of the assembly processes that have shaped *Cyclophorus* communities among northern Vietnam's limestone karsts.

## 2 MATERIAL AND METHODS

### 2.1 Sampling design

**Figure 1:**
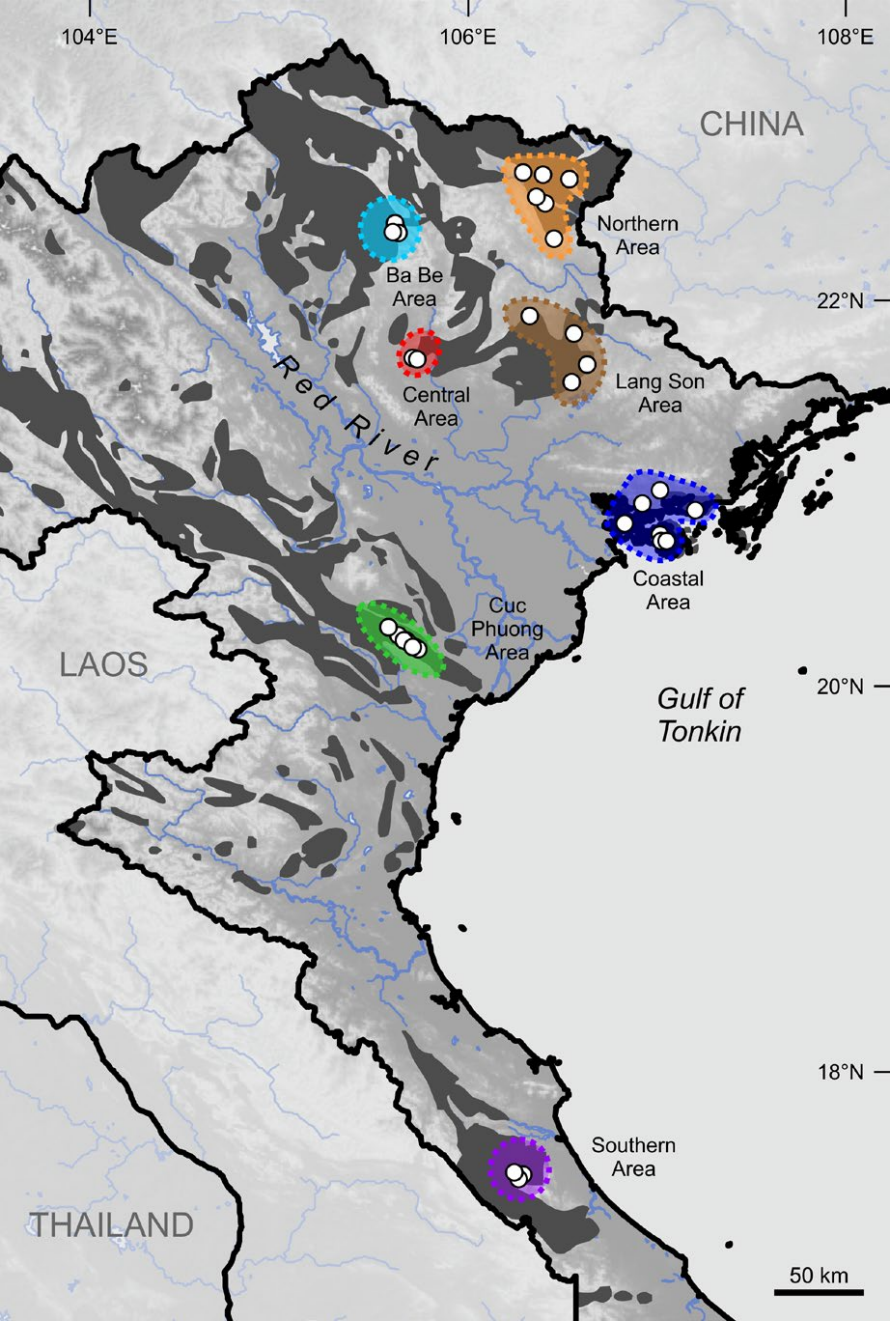
Map of northern Vietnam including the seven studied karst areas (colored; Ba Be, Central, Coastal, Cuc Phuong, Lang Son, Northern, and Southern Areas) and individual sampling localities (white dots) of the material used for the analyses of community structure. Limestone karst is labeled in dark gray (distribution according to Dang, 1996)

For studying *Cyclophorus* communities in limestone karst habitats in northern Vietnam, study areas were defined a priori based on the following three criteria: (1) Each area must be located in a region where limestone karst is present (based on Dang (1996) and satellite imagery available via Google Earth Pro 7.1.8.3036; Google Inc., Mountain View, CA, USA); (2) each area must be characterized by a distinct and homogeneous *Cyclophorus* fauna (peripheral zones with overlapping distribution ranges of other *Cyclophorus* lineages, which were absent from the core areas, were excluded); and (3) for each area, a comprehensive number of *Cyclophorus* samples must be present in the NHM collections, to ensure that the entire community has been sampled. Altogether, seven karst areas have been selected (Ba Be, Central, Coastal, Cuc Phuong, Lang Son, Northern, and Southern Areas; see Figure 1), all of them geographically distant (>40 km distance) from each other.

For this study, the entire collections of *Cyclophorus* spp. at the NHM were examined. Recent and historical collections of dry shells and fluid‐preserved specimens, including all relevant type material (see Nantarat et al., 2014a), were examined thoroughly and all material that had locality information suitable for assigning it to one of the studied karst areas was included in the primary dataset. Given that the taxonomic information available for Vietnamese *Cyclophorus* spp. is very limited and that a revision of the group was beyond the scope of the present study, all studied specimens were referred to as “*Cyclophorus* sp.”. Instead of using species names for classification, the studied groups were defined based on relative divergence times from the Bayesian phylogeny (see below and results) as an arbitrary, but objective, criterion. Accordingly, all clades of Vietnamese *Cyclophorus* with a node depth of >0.04 substitutions per site in the phylogeny were regarded as separate “lineages”. The studied specimens were classified into a total of 15 lineages (including the morphologically distinct lineage 15 from the Northern Area for which no molecular data could be obtained as only one single empty shell was available). All specimens belonging to the same lineage showed a relatively consistent shell morphology, and all available specimens from the study areas could be clearly assigned to one of the lineages.

From the primary dataset, which contained all specimens from the studied karst areas that were available in the NHM collections, three specimens per lineage and karst area were included in the molecular dataset and five specimens per lineage and per karst area in the morphological dataset (similar specimen numbers have been used in other studies on animal community assembly; Geheber & Geheber, 2016; Ingram & Shurin, 2009), as far as available in the collections (see Table S1). Only fully grown (determined by their thickened aperture) individuals of both sexes were included. The specimens used for the molecular and morphological datasets were from a total of 40 individual sampling localities (Table S1, Figure 1). All specimens from the molecular dataset were part of the morphological dataset. As it remained doubtful whether the single shell of lineage 15 (specimen 15/01) belongs to a taxon that is really established in the Northern Area and as no molecular data were available for this lineage, it was only included in an additional version of the morphological dataset.

### 2.2 DNA isolation, PCR, and sequencing

The molecular dataset was based on 50 Vietnamese *Cyclophorus* specimens (all sequenced individuals in Table S1). DNA isolation was carried out using ethanol‐preserved foot tissue of individual gastropods and the protocol of Winnepenninckx, Backeljau, and De Wachter (1993). A 658‐bp fragment of the mitochondrial cytochrome *c* oxidase subunit I (COI) gene, a 498–511‐bp fragment of the mitochondrial 16S rRNA gene, and a 583–592‐bp fragment of the nuclear 28S rRNA gene were amplified. Primers for the COI gene were LCO1490 and HCO2198 (Folmer, Black, Hoeh, Lutz, & Vrijenhoek, 1994), primers for the 16S rRNA gene were 16Sar‐L and 16Sbr‐H (Palumbi et al., 1991), and primers for the 28S rRNA gene were 28SF4 and 28SR5 (Morgan et al., 2002). PCR conditions for COI were as follows: 120 s at 94°C, 36 cycles (30 s at 94°C, 120 s at 42°C, and 120 s at 72°C), and 300 s at 72°C (Nantarat et al., 2014b). PCR conditions for 16S and 28S were as follows: 300 s at 94°C, 29 cycles (30 s at 95°C, 30 s at 52°C, and 30 s at 72°C), and 300 s at 72°C. DNA sequencing (forward and reverse) was carried out on an AB 3730XL DNA Analyzer (Applied Biosystems, Waltham, MA, USA) using the BigDye Terminator v3.1 Cycle Sequencing Kit (Applied Biosystems). Heterozygous insertions or deletions in the 28S dataset were resolved using Mixed Sequence Reader (Chang et al., 2012). All heterozygous sites (<0.1% of all positions in 28S) were regarded as missing data. All sequences have been deposited at GenBank (Table S1).

### 2.3 Sequence alignment and phylogenetic analysis

Sequence data from 32 additional *Cyclophorus* specimens and three outgroup taxa (Table S2) were taken from the literature (Nantarat et al., 2014b; Nantarat, Wade, Jeratthitikul, Sutcharit, & Panha, 2014c; see there for locality details of this material). The sequences of the protein‐coding COI gene were aligned using CLUSTAL W (Thompson, Higgins, & Gibson, 1994) in BIOEDIT version 7.2.6 (Hall, 1999). The sequences of the noncoding 16S and 28S genes were aligned using the Q‐INS‐i strategy (Katoh & Toh, 2008), which considers the secondary structure of RNA, with default settings in MAFFT version 7 (Katoh & Standley, 2013). The removal of identical sequences from the primary alignment of 85 sequences resulted in a final dataset of 79 unique sequences. The COI (codon positions 1/2 and 3 separately), 16S, and 28S datasets were tested for substitution saturation using the entropy‐based method of Xia, Xie, Salemi, Chen, and Wang (2003) implemented in DAMBE 6.4.67 (Xia, 2017; Xia & Lemey, 2009). Under the assumption of a symmetrical tree, the test showed little saturation for all datasets. Even under the unlikely assumption of an asymmetrical tree, the COI codon positions 1/2 as well as 16S, and 28S showed little saturation, and only the COI codon position 3 was rated as useless.

Best fit models of sequence evolution were determined by applying the corrected Akaike information criterion in jModelTest 2.1.9 (Darriba, Taboada, Doallo, & Posada, 2012). Models suggested were GTR+I+G for COI and 16S, and TIM2+I+G for 28S. Bayesian inference was performed utilizing MrBayes 3.2.6 (Ronquist et al., 2012). As TIM2+I+G is not implemented in MrBayes, GTR+I+G was used instead as the closest overparameterized model (Huelsenbeck & Rannala, 2004). Two parallel runs with four chains (one cold and three heated) were carried out for 5,000,000 generations (50,000 trees). Checking the runs using Tracer v1.6.0 (Rambaut, Suchard, Xie, & Drummond, 2014), they showed high effective sample size values (>300 for all parameters) and smooth frequency plots. For the ultrametric consensus tree, 10% of the sampled trees were discarded as burn‐in.

### 2.4 Morphometric analysis

The morphological dataset was based on 83 Vietnamese *Cyclophorus* specimens (all individuals in Table S1 except 15/01). Shells were digitally photographed in standardized orientation (apertural view, the central axis parallel to the ground), and shell outlines were traced using Corel Draw X7 (Corel Corporation, Ottawa, Canada). Outline images were compiled using tpsUtil version 1.70 (Rohlf, 2016). Subsequently, tpsDig 2 version 2.30 (Rohlf, 2017) was used to create 200 semilandmarks along the outline following Van Bocxlaer and Schultheiß (2010). The first landmark marked the widest point of the aperture on the right side of the outline. In cases where parts of the aperture were missing, the outline was interpolated. An additional version of the morphometric dataset was created by including lineage 15 (specimen 15/01); all subsequent steps were carried out for both of these datasets separately.

A Procrustes transformation was performed in PAST version 3.15 (Hammer, Harper, & Ryan, 2001) to remove variation in the orientation and position of the landmarks. The shell size (centroid size) was kept, as it has been shown to be an ecologically highly relevant trait (Chiba, 2004; Lee & Silliman, 2006; Schmera & Baur, 2014). In addition, a further set of datasets was created by removing the size of specimens. Average landmark data were calculated for all *Cyclophorus* lineages. In order to take the morphological variability among populations and thus potential effects of different selective pressures in different areas into account (Jung, Violle, Mondy, Hoffmann, & Muller, 2010; see also discussion in Gerhold et al., 2015), averages for each population were calculated separately for lineages occurring in multiple karst areas. With these average landmarks, a principal component analysis (PCA, relative warps) for 2D landmark data was carried out in PAST version 3.15. On the basis of the broken‐stick model (Jackson, 1993), only the first principal component (PC1) was chosen for further analysis.

### 2.5 Analyses of community structure

To analyze potential patterns of phylogenetic overdispersion or clustering, first, a matrix of cophenetic distances between representatives of the different *Cyclophorus* lineages (based on the Bayesian phylogeny and rounded to 4 digits; see results) was generated, which was then used to calculate the mean nearest taxon distance (MNTD) of communities. The MNTD was calculated for the observed communities and for 9,999 sets of communities that were randomly created using the independent swap algorithm (Gotelli, 2000). This algorithm maintains the total occurrence frequency of taxa (among all karst areas) as well as the richness of taxa in individual communities. Afterward, the standardized effect size (SES) of the MNTD (Webb, 2000; Webb et al., 2002) was determined for all communities by comparing the observed MNTD with the randomly created MNTDs and respective *p*‐values were calculated (but note that the focus of this study was overall patterns among all studied communities rather than significance at particular areas). SES values greater than zero indicated phylogenetic overdispersion and values smaller than zero indicated phylogenetic clustering.

To test for overdispersion or clustering of shell morphologies, distance matrices were calculated from the PC1 values of the morphometric analysis. Using the same approach as for the phylogenetic dataset, the mean nearest trait distance (analogous to the mean nearest taxon distance, with trait distance being used instead of phylogenetic distance) was calculated for the observed communities and 9,999 sets of random communities that were created with the independent swap algorithm. Communities with co‐occurring populations from the same lineage were excluded from the randomized dataset. Afterward, the SES of the mean nearest trait distance and *p*‐values were calculated for all communities. SES values greater than zero indicated morphological overdispersion and values smaller than zero indicated morphological clustering.

The analyses of community structure were carried out using R version 3.4.0 (R Core Team 2017) with the package Picante 1.6‐2 (Kembel et al., 2010).

## 3 RESULTS

**Figure 2:**
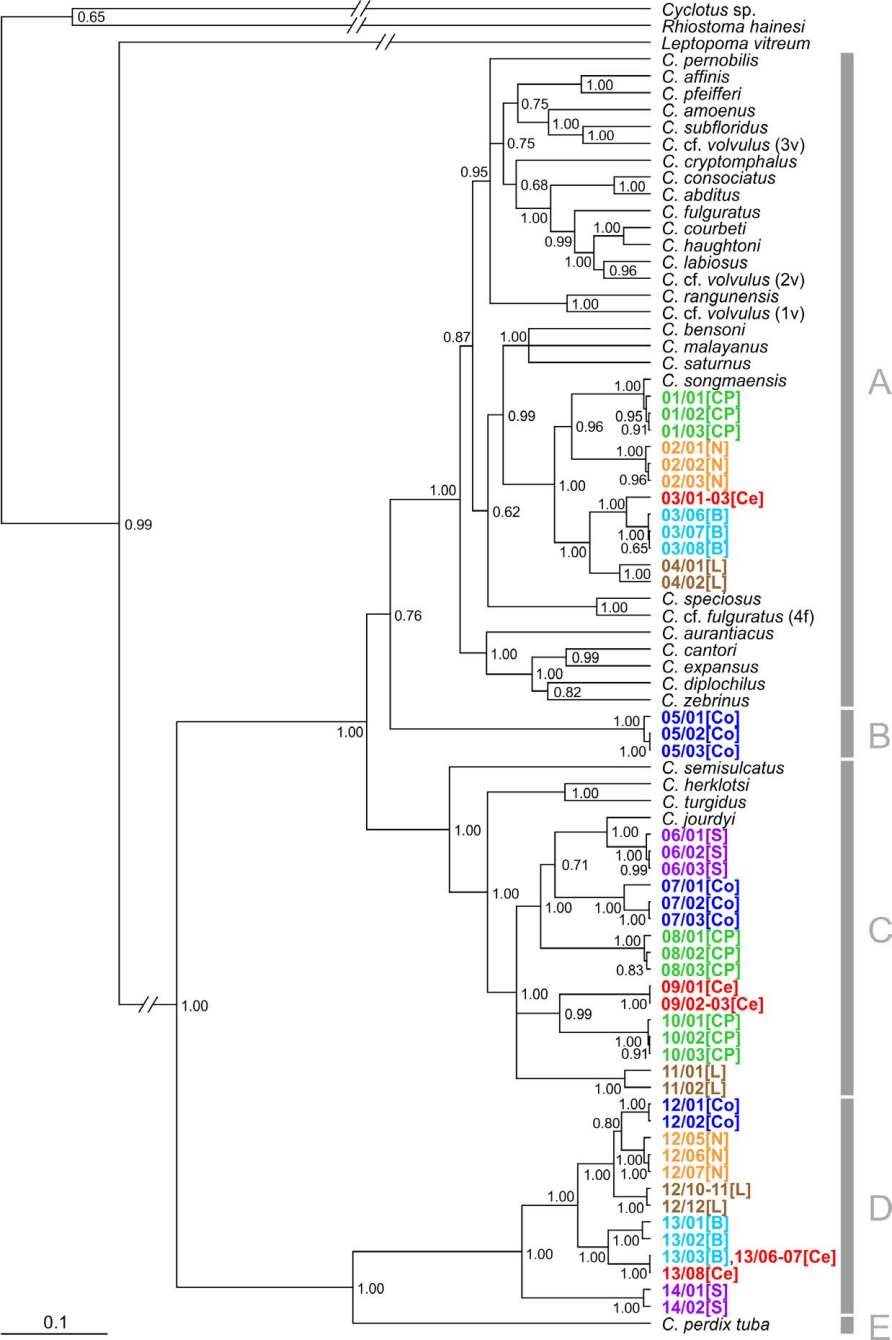
Consensus ultrametric Bayesian phylogeny of *Cyclophorus* spp. based on the COI, 16S, and 28S genes. Bayesian posterior probabilities are given next to the respective node. The scale bar indicates substitutions per site according to the applied model of sequence evolution. Major clades are labeled (A–E), and specimen codes of all individuals sharing a certain haplotype are provided next to the respective tree tip. The first number of the specimen code indicates the respective lineage (01–14), and specimens are labeled and color‐coded according to the different karst areas (B, Ba Be; Ce, Central; Co, Coastal; CP, Cuc Phuong; L, Lang Son; N, Northern; S, Southern). For detailed specimen information, see Table S1

The Bayesian phylogeny revealed that four well‐supported (Bayesian posterior probabilities (BPP) of 1.00) major clades of *Cyclophorus* spp. are distributed in northern Vietnam (clade A, B, C, and D; Figure 2). Out of the 14 *Cyclophorus* lineages (clades with a node depth of >0.04 substitutions per site; all supported with BPP = 1.00) within the phylogeny, four were part of clade A, one lineage formed clade B, six were part of clade C, and three were part of clade D. Besides the Vietnamese taxa, clade A included *Cyclophorus* species from Thailand and Laos, Clade C included species from peninsular Malaysia and Japan, and the peninsular Malaysian *Cyclophorus perdix tuba* formed a separate major clade (clade E).

**Figure 3:**
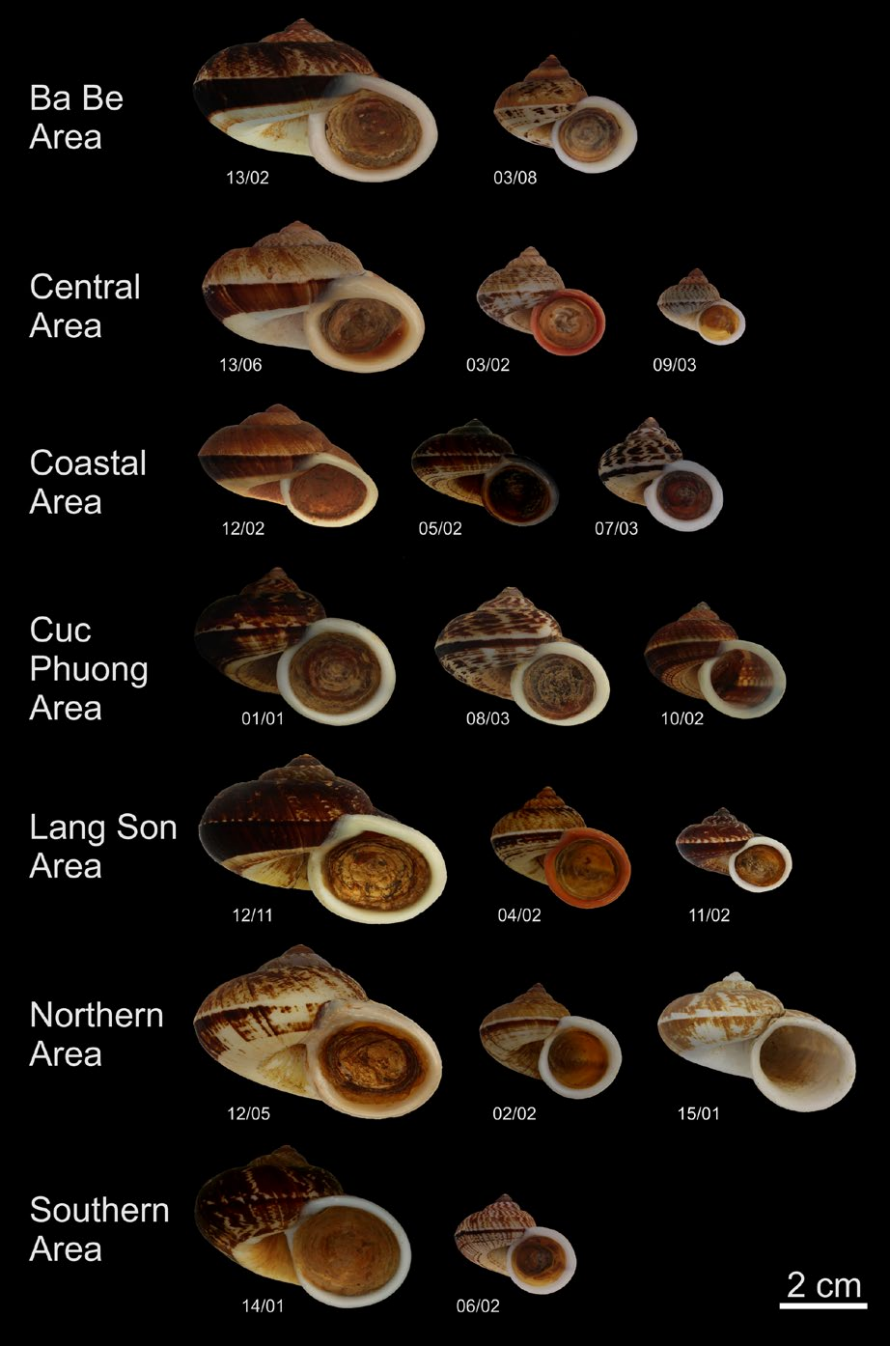
Individuals of all *Cyclophorus* lineages from each of the studied karst areas. Note, that individuals, which show an average morphology within the range of intra‐lineage/area variation, are depicted. All specimens displayed were part of the phylogenetic and morphological datasets, except lineage 15 (specimen 15/01), which was only part of an additional version of the morphological dataset

Two to three different *Cyclophorus* lineages were found to co‐occur in each of the studied karst areas (Figure 3). The composition of individual communities was always unique. Only in the Ba Be and Central Areas, two shared lineages were present (lineage 03 and 13) and one shared lineage was found in the Coastal, Northern, and Lang Son Areas (lineage 12).

**Figure 4:**
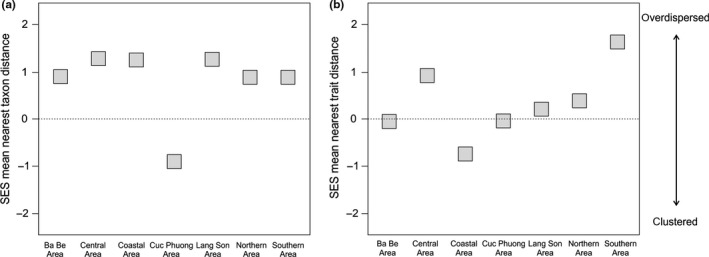
Standardized effect size (SES) of mean nearest taxon distance (a; based on the phylogenetic dataset) and SES of mean nearest trait distance (b; based on the morphological dataset) of *Cyclophorus* communities from seven studied karst areas. Positive SES values indicate overdispersion and negative SES values indicate clustering

The analysis of phylogenetic community structure revealed in six of seven studied karst areas a pattern of overdispersion (Figure 4a, Table 1). Only in the Cuc Phuong Area, a pattern of phylogenetic clustering was found.

**Table 1:** Results of community structure analyses of *Cyclophorus* communities from seven studied karst areas including the standardized effect size (SES) of mean nearest taxon distance and respective *p*‐values (two‐tailed *t*‐test) for the phylogenetic dataset, and the SES of mean nearest trait distance and respective *p*‐values (two‐tailed *t*‐test) for the morphological dataset

Area	SES (mean nearest taxon distance)	*p*‐Value for SES of mean nearest taxon distance	SES (mean nearest trait distance)	*p*‐Value for SES of mean nearest trait distance
Ba Be	0.89	.25	−0.05	.54
Central	1.28	.15	0.91	.19
Coastal	1.25	.15	−0.75	.26
Cuc Phuong	−0.90	.18	−0.04	.51
Lang Son	1.26	.15	0.20	.42
Northern	0.88	.26	0.39	.34
Southern	0.88	.26	1.63	.09

Positive SES values indicate overdispersion and negative SES values indicate clustering.

For the morphological dataset, the first principal component (PC1), which was probably primarily associated with shell size, explained 95.30% of the total variance (see Table S3 for PC scores). The analysis of morphological community structure showed in four of seven karst areas a pattern of overdispersion (Figure 4b, Table 1). These were the Central, Lang Son, Northern, and Southern Areas, while in the Southern Area, the most pronounced pattern was detected. The communities in three areas were morphologically clustered: in the Ba Be and Cuc Phuong Areas, in which the values were very close to a random distribution, and in the Coastal Area. The inclusion of lineage 15 in the morphological analysis (PC1 explained 93.58%; PC scores in Table S3) had minor effects on patterns of community structure among areas; only in the Northern Area (where this specimen was found), the community structure changed from being overdispersed to clustered (close to a random distribution) when lineage 15 was included (Figure S1a, Table S4).

When size was discarded in the analyses (PC1 explained 76.92%; PC scores in Table S3), only the communities in the Central and Lang Son Areas were overdispersed (Figure S1b, Table S4). The communities in all other areas were clustered, with those from the Cuc Phuong and Southern Areas showing the most pronounced patterns. The inclusion of lineage 15 in this dataset (PC1 explained 80.02%; PC scores in Table S3) had minor effects on the patterns of community structure in all areas, but a pronounced effect on the Northern Area community. The community was slightly clustered when lineage 15 was excluded, but clearly overdispersed when it was included (Figure S1c, Table S4).

## 4 DISCUSSION

In the present study, the main processes involved in the assembly of land snail biodiversity on limestone karsts were systematically investigated for the first time. Our analyses revealed, for six of the seven studied *Cyclophorus* communities, a pattern of phylogenetic overdispersion. Four of the phylogenetically overdispersed communities also showed a pattern of morphological overdispersion. In the respective areas (Central, Lang Son, Northern, and Southern), all co‐occurring taxa were more distantly related and morphologically more dissimilar than expected by chance. Such a pattern indicates that competition among the different *Cyclophorus* lineages rather than filtering due to geographical factors is the major process that has shaped these communities. Competition among different lineages represents the most parsimonious explanation for the pattern found, while other processes, which may potentially lead to a pattern of overdispersion (such as effects of specific pathogens or intraguild egg predation; Cavender‐Bares et al., 2009; Polis & Holt, 1992), cannot be excluded.

The additional analysis focusing only on shell shape (discarding the size of specimens) revealed a pattern of morphological overdispersion for only two of the seven studied communities. However, as different lineages with distinct size classes have been found in almost each area (see Figure 3), shell size is probably a very relevant character for limiting morphological similarity in *Cyclophorus* communities, while shell shape alone may be less relevant. The largest size class was in most cases formed by lineages of clade D, with the exception of that in the Cuc Phuong Area (see discussion below). The most widespread group within clade D, lineage 12, showed pronounced differences in shell form in different karst areas, which presumably reflect adaptations to local environmental conditions. The medium and small size classes were formed by lineages of clades A, B, and C. Among them, highly similar shells in different karst areas belonging to distantly related lineages (e.g., lineages 03, 07, and 10) could have resulted from convergent evolution.

As the ecological differences between the various *Cyclophorus* lineages are virtually unknown, the exact mechanisms of competition among them remain obscure. The pronounced pattern of different size classes, however, indicates that size is involved in limiting the competition between lineages. The shell size of land snails can be strongly related to their habitat and ecology (Goodfriend, 1986). Species with different shell sizes may, for example, use different microhabitats in the same area, as shown for camaenid land snails from the Bonin Islands, where large and heavy shelled taxa are restricted to the ground, while smaller taxa can crawl on shrubs, branches, and leaves (Chiba, 2004). In *Cyclophorus* spp., however, there is no evidence for a substantial vertical habitat partitioning as specimens from differently sized lineages were mainly found on the ground, even though juvenile and smaller individuals have been occasionally observed climbing actively over leaves and twigs of low lying vegetation (P.V. and K.C.M. von Oheimb, and F. Naggs, personal observation). Size differences may be also related to the ability to explore different food resources: Smaller snails might reach food in narrower gaps and crevices. Steneck and Watling (1982) argued that body size is one of the most important characters reflecting the diets of mollusks. A certain size of the feeding apparatus (which can be linked to body size; see Meirelles & Matthews‐Cascon, 2003; Van Cleave & McDavid Richey, 1936), for example, can allow gastropods to feed on plant material of specific size or toughness (Norton & Manley, 1990; Thomas, Nwanko, & Sterry, 1985). The examination of further morphological traits in Vietnamese *Cyclophorus* spp., such as the shape of the radula, or studies on specimens’ gut content could be used to reveal the ecological requirements of different *Cyclophorus* lineages, which could help to determine their ecological niches and provide further evidence on the type and degree of competition among them. Observations in the field or laboratory experiments could be used to reveal which microhabitats they use, what limiting resources are, and how they interact.

Patterns that were contradictory between the phylogenetic and the morphological dataset have been found in two of the phylogenetically overdispersed communities. While the Ba Be Area community was slightly morphologically clustered, close to a random distribution, the Coastal Area community showed a clear pattern of morphological clustering. The pattern found in the Coastal Area indicates that interspecific competition (by excluding phylogenetically similar taxa) had primarily shaped the community and that similar ecological adaptations have obscured the respective pattern in shell morphology. Specimens in this area were generally smaller and more similar in size and shape than those in other areas (see Figure 3). The Coastal Area is located around Ha Long Bay at the Gulf of Tonkin and includes Cat Ba Island, which is completely surrounded by seawater, as well as parts of the mainland, which adjoin the sea. In contrast to the other studied karst areas, individual limestone habitats in the Coastal Area are smaller and more fragmented. In addition, the environmental conditions of the Coastal Area differ from those inland: Cat Ba Island, for example, is characterized by warmer winters and cooler summers and is more exposed to typhoons (Do, 2014; Nguyen, Tran, & Dinh, 2010). The Ha Long Bay region also includes far smaller islands than Cat Ba Island, which were not included in our study area. Interestingly, dry material from one of these smaller islands was available in the NHM collections (coordinates: 20.8808, 107.1272; NHMUK 19991467), with shells resembling specimens from lineage 05 in terms of shell form but significantly smaller. These specimens may thus represent a dwarfed variety of this lineage. Such a phenomenon is also known from other land snails. Páll‐Gergely et al. (2015) noticed that populations of *Halongella schlumbergeri* (Plectopylidae) tend to have smaller shell widths on smaller islands in the Ha Long Bay region. Different selective pressures, caused by fewer resources or harsher environmental conditions on the smaller islands (Vermeulen & Maassen, 2003), may limit the shell size.

The community in the Cuc Phuong Area was the only one, which showed both a phylogenetic and a morphological clustering (the latter close to a random distribution). The pattern of phylogenetic clustering was mainly caused by the absence of a member of clade D, while a lineage of this phylogenetically very distinct group was part of all other communities. Instead, two lineages from clade C were present in the Cuc Phuong Area (lineages 08 and 10). Filtering due to geographical factors could thus have influenced this community and prevented the establishment of a member of clade D in this area. Habitat filtering, however, would require that certain environmental factors differ from all other karst areas, for which we have no obvious evidence. An alternative explanation would be that dispersal barriers, such as the Red River that is located north of the region (Figure 1), may have prevented the colonization of the Cuc Phuong Area by clade D from regions north of the river. This river has also been found to be associated with a biogeographical barrier for other organisms (e.g., Bain & Hurley, 2011; Zhang & Li, 2013). However, another member of clade D, lineage 14, does occur further south, in the Southern Area. It is quite striking that specimens of the lineage with the largest shells present in the Cuc Phuong Area (lineage 01, which belongs to clade A; note that this lineage also includes a specimen from Nantarat et al. (2014b) that was determined as *Cyclophorus songmaensis*) resemble specimens of clade D in shell morphology (see Figure 3); a pattern that may have been caused by convergent evolution.

Lineage 15, which was found in the Northern Area, is morphologically distinct from all other lineages. However, as this lineage is only represented by a single empty shell in the collection and was not found regularly co‐occurring with other lineages from this area, it may represent a rare record outside of its distribution range. Further studies are thus needed to clarify the exact distribution of lineage 15.

The present study shows how museum collections can be used to examine community assembly, if model systems and research questions are chosen carefully based on the available material. For studying communities of taxa that are less conspicuous or more challenging to collect than *Cyclophorus* spp., however, already established collections may not provide sufficient data. In such cases, additional sampling based on standardized protocols should be carried out for validation. We could show that, in addition to more recent, ethanol‐preserved collections, material from historical collections can be included in such a dataset when the respective locality information is reviewed very carefully. However, although we focused on a very prominent taxon in one of the largest land snail collections in the world, only relatively few karst areas were sufficiently sampled to use them for the analyses. Given that the NHM's collections of Vietnamese land snails do not include information about the exact habitats of taxa, which could reveal factors that are potentially involved in competition among the lineages (e.g., ecological requirements or food preferences), the present study underlines the importance of recording environmental parameters during sampling campaigns.

The findings of our study highlight the island‐like character of Vietnam's karst areas, which are inhabited by unique, mainly competition‐driven communities of *Cyclophorus* spp. The detected patterns and processes, which could also be present in many other groups of karst organisms that share similar habitat requirements and dispersal abilities, underline the important role of these systems for the development and maintenance of the country's rich biodiversity. Due to their discrete nature, island‐like systems are very suitable for studies of community assembly (Emerson & Gillespie, 2008; Losos & Ricklefs, 2009). However, comparisons among different island‐like systems have to be made very carefully, as spatial (patterns of clustering and overdispersion can be a matter of scale) and temporal characteristics (an initial clustering can change into overdispersion over time) strongly influence detected patterns (Emerson & Gillespie, 2008). Nevertheless, some common patterns derive from studies on oceanic islands, for example, the repeated evolution of different co‐occurring ecomorphs on different islands. This was shown for various taxa, for example, *Tetragnatha* spiders from the Hawaiian Islands (Gillespie, 2004), *Anolis* lizards from the Greater Antilles (Losos, Jackman, Larson, de Queiroz, & Rodríguez‐Schettino, 1998), and *Bulimulus* land snails and Darwin's finches from the Galapagos Islands (Grant & Grant, 1979; Parent & Crespi, 2006). Such findings were mainly made in relatively old, large, and remote island systems and often involve a phylogenetic clustering of respective communities due to in situ speciation. However, on smaller, younger, and/or less remote islands, such as the Cocos Island (a small island north of the Galapagos Islands) or the Lesser Antilles, speciation events generally occurred less frequently and endemics are more closely related to mainland species (Emerson & Gillespie, 2008; Losos & Ricklefs, 2009). Although we found different size classes among co‐occurring *Cyclophorus* lineages, which could reflect specific ecological adaptations, we never found sister group relationships between lineages from the same area (not even in the Cuc Phuong Area, where two lineages from clade C occur). Thus, dispersal processes among karsts probably play a much more important role than intrakarst speciation; a pattern which generally resembles those found on less remote oceanic islands. In contrast to *Cyclophorus* spp., a number of highly specialized karst organisms depend on the limestone‐rich environment to a much higher degree and are probably much less effective dispersers (such as cave organisms and microgastropods; Deharveng & Bedos, 2012; Jochum et al., 2014). In these organisms, communities are expected to be phylogenetically more clustered within the same karst area due to processes of in situ speciation and a higher impact of dispersal barriers. Future research should focus on different groups of limestone‐bound taxa that potentially differ in their dispersal abilities in order to detect parameters that further influence community assembly among Vietnam's limestone karsts.

## CONFLICT OF INTEREST

None declared.

## AUTHORS CONTRIBUTION

PVvO, KCMvO, and FN designed the study. TH, TVD, HVL, JA, SVP, and FN acquired the majority of the material/data. PVvO and KCMvO carried out the analyses. PVvO and KCMvO wrote the paper and included comments and suggestions from all other authors.
