# Supplementary material for: Competition matters: Determining the drivers of land snail community assembly among limestone karst areas in northern Vietnam
Source: Ecol Evol. 2018 Mar 26;8(8):4136–49. doi: 10.1002/ece3.3984 (PMC5916308; doi:10.1002/ece3.3984)
Supplement: Supplementary file 1 [file ECE3-8-4136-s001.pdf]

Supporting information

Table S1. List of studied individuals including specimen and locality details. The first number of the specimen code indicates the respective lineage (01–15). GenBank accession numbers are given for all sequenced individuals. The voucher material is deposited at the Natural History Museum, London.

Specimen code	Original sample no.	Area	Latitude	Longitude	Locality	COI GenBank accession no.	16S GenBank accession no.	28S GenBank accession no.	Registration no. (NHMUK)
01/01	119(2013)a	Cuc Phuong	20.35878	105.57733	Ninh Binh, Cuc Phuong National Park	MG720888	MG720938	MG720988	20140299
01/02	149(2013)a	Cuc Phuong	20.29562	105.66362	Ninh Binh, Cuc Phuong National Park	MG720890	MG720940	MG720990	20140329/1
01/03	328(2013)a	Cuc Phuong	20.29358	105.66873	Ninh Binh, Cuc Phuong National Park	MG720895	MG720945	MG720993	20140509
01/04	124(2013)a	Cuc Phuong	20.25053	105.71515	Ninh Binh, Cuc Phuong National Park				20140304
01/05	309(2013)a	Cuc Phuong	20.359	105.577	Ninh Binh, Cuc Phuong National Park				20140490
02/01	VN050	Northern	22.5525	106.39639	Cao Bang, Hong Nam commune	MG720904	MG720954	MG721002	20170420
02/02	VN040	Northern	22.70617	106.28149	Cao Bang, Nguyen Hue commune	MG720899	MG720949	MG720997	20170413
02/03	VN041	Northern	22.58867	106.35206	Cao Bang, Hong Nam commune	MG720900	MG720950	MG720998	20170414
02/04	D3(2011)a	Northern	22.69657	106.37747	Cao Bang, Quoc Dan commune				20110547
02/05	VN025	Northern	22.69657	106.37747	Cao Bang, Quoc Dan commune				20170408
03/01	A5(2011)g	Central	21.75523	105.70745	Thai Nguyen, Dong Dat commune	MG720863	MG720913	MG720963	20110476/7
03/02	A5(2011)h	Central	21.75523	105.70745	Thai Nguyen, Dong Dat commune	MG720864	MG720914	MG720964	20110476/8
03/03	A5(2011)i	Central	21.75523	105.70745	Thai Nguyen, Dong Dat commune	MG720865	MG720915	MG720965	20110476/9
03/04	A(2011)h	Central	21.75523	105.70745	Thai Nguyen, Dong Dat commune				20170405
03/05	A5(2011)k	Central	21.75523	105.70745	Thai Nguyen, Dong Dat commune				20110476/11
03/06	B6(2011)b	Ba Be	22.44617	105.6174	Bac Kan, Ba Be National Park	MG720871	MG720921	MG720971	20110488/2
03/07	B31(2011)c	Ba Be	22.40923	105.62855	Bac Kan, Ba Be National Park	MG720873	MG720923	MG720973	20110513/3
03/08	B31(2011)d	Ba Be	22.40923	105.62855	Bac Kan, Ba Be National Park	MG720874	MG720924	MG720974	20110513/4
03/09	VN117	Ba Be	22.40761	105.611	Bac Kan, Ba Be National Park				20170429
03/10	VN106	Ba Be	22.44685	105.6172	Bac Kan, Ba Be National Park				20170428
04/01	VN037	Lang Son	21.8787	106.54739	Lang Son, Vinh Lai commune	MG720898	MG720948	MG720996	20170411
04/02	VN042	Lang Son	21.72352	106.60944	Lang Son, Mai Sao commune	MG720901	MG720951	MG720999	20170415
04/03	VN038	Lang Son	21.8787	106.54739	Lang Son, Vinh Lai commune				20170412
04/04	VN043	Lang Son	21.72352	106.60944	Lang Son, Mai Sao commune				20170416
04/05	VN044	Lang Son	21.72352	106.60944	Lang Son, Mai Sao commune				20170417
05/01	G6(2011)e	Coastal	20.96638	107.1661	Quang Ninh, Ha Long, Ha Phong ward	MG720881	MG720931	MG720981	20110609/5
05/02	G6(2011)a	Coastal	20.96638	107.1661	Quang Ninh, Ha Long, Ha Phong ward	MG720879	MG720929	MG720979	20110609/1
05/03	G6(2011)d	Coastal	20.96638	107.1661	Quang Ninh, Ha Long, Ha Phong ward	MG720880	MG720930	MG720980	20110609/4
05/04	G6(2011)b	Coastal	20.96638	107.1661	Quang Ninh, Ha Long, Ha Phong ward				20110609/2
05/05	VN210	Coastal	20.8	107	Hai Phong, Cat Ba National Park				20170431
06/01	88(2012)a	Southern	17.54147	106.23532	Quang Binh, Phong Nha-Ke Bang National Park	MG720882	MG720932	MG720982	20130894/1
06/02	102(2012)c	Southern	17.50317	106.26045	Quang Binh, Phong Nha-Ke Bang National Park	MG720883	MG720933	MG720983	20130904/3
06/03	117(2012)a	Southern	17.52843	106.27782	Quang Binh, Phong Nha-Ke Bang National Park	MG720884	MG720934	MG720984	20130915
06/04	126(2012)b	Southern	17.50317	106.26045	Quang Binh, Phong Nha-Ke Bang National Park				20130921/2
06/05	154(2012)b	Southern	17.50317	106.26045	Quang Binh, Phong Nha-Ke Bang National Park				20130942/2
07/01	F4(2011)b	Coastal	21.06693	106.98883	Quang Ninh, Son Duong commune	MG720876	MG720926	MG720976	20110603/2
07/02	G5(2011)b	Coastal	20.96638	107.1661	Quang Ninh, Ha Long, Ha Phong ward	MG720877	MG720927	MG720977	20110608/2
07/03	G5(2011)c	Coastal	20.96638	107.1661	Quang Ninh, Ha Long, Ha Phong ward	MG720878	MG720928	MG720978	20110608/3
07/04	VN203	Coastal	20.84	106.99	Hai Phong, Cat Ba National Park				20170430
07/05	VN246	Coastal	20.9**	106.8**	"Haiphong"				20170433
08/01	323(2013)a	Cuc Phuong	20.25038	105.71478	Ninh Binh, Cuc Phuong National Park	MG720894	MG720944	MG720992	20140504/1

08/02	150(2013)e	Cuc Phuong	20.29562	105.66362	Ninh Binh, Cuc Phuong National Park	MG720891	MG720941	MG720991	20140330/5
08/03	198(2013)a	Cuc Phuong	20.30358	105.65478	Ninh Binh, Cuc Phuong National Park	MG720892	MG720942	n.a.	20140378/1
08/04	198(2013)b	Cuc Phuong	20.30358	105.65478	Ninh Binh, Cuc Phuong National Park				20140378/2
08/05	322(2013)b	Cuc Phuong	20.27653	105.6799	Ninh Binh, Cuc Phuong National Park				20140503/2
09/01	H9(2009)a	Central	21.7551	105.7073	Thai Nguyen, Dong Dat commune	MG720861	MG720911	MG720961	20170403
09/02	A5(2011)l	Central	21.75523	105.70745	Thai Nguyen, Dong Dat commune	MG720867	MG720917	MG720967	20110476/12
09/03	A5(2011)j	Central	21.75523	105.70745	Thai Nguyen, Dong Dat commune	MG720866	MG720916	MG720966	20110476/10
09/04	A3(2011)b	Central	21.75523	105.70745	Thai Nguyen, Dong Dat commune				20110474/2
09/05	A3(2011)c	Central	21.75523	105.70745	Thai Nguyen, Dong Dat commune				20110474/3
10/01	313(2013)a	Cuc Phuong	20.359	105.577	Ninh Binh, Cuc Phuong National Park	MG720893	MG720943	n.a.	20140494
10/02	96(2013)d	Cuc Phuong	20.28845	105.66767	Ninh Binh, Cuc Phuong National Park	MG720887	MG720937	MG720987	20140276/4
10/03	126(2013)b	Cuc Phuong	20.25053	105.71515	Ninh Binh, Cuc Phuong National Park	MG720889	MG720939	MG720989	20140306/2
10/04	151(2013)e	Cuc Phuong	20.29562	105.66362	Ninh Binh, Cuc Phuong National Park				20140331/5
10/05	V146(2008)b	Cuc Phuong	20.258974	105.706492	Ninh Binh, Cuc Phuong National Park				20160707
11/01	VN073	Lang Son	21.71884	106.6127	Lang Son, Mai Sao commune	MG720907	MG720957	MG721005	20170423
11/02	VN074	Lang Son	21.97243	106.31021	Lang Son, Mong An commune	MG720908	MG720958	MG721006	20170424
11/03	VN247	Lang Son	21.63*	106.53*	"Than-Moi, Tonkin"				1901.12.12.67
12/01	V159(2008)a	Coastal	20.8	107	Hai Phong, Cat Ba National Park	MG720859	MG720909	MG720959	20160724
12/02	V159(2008)b	Coastal	20.8	107	Hai Phong, Cat Ba National Park	MG720860	MG720910	MG720960	20160725
12/03	VN245	Coastal	21**	106.9**	"Montagnes des environs d'Haiphong (Tonkin)" [Mountains in the environs of Haiphong (Tonkin)]				1893.12.8.43
12/04	VN244	Coastal	20.8008	107.0222	Hai Phong, Cat Ba National Park				19991459
12/05	VN046	Northern	22.67799	106.52164	Cao Bang, Cai Bo commune	MG720903	MG720953	MG721001	20170419
12/06	VN004	Northern	22.6781	106.5228	Cao Bang, Cai Bo commune	MG720896	MG720946	MG720994	20170407
12/07	VN036	Northern	22.6781	106.5228	Cao Bang, Cai Bo commune	MG720897	MG720947	MG720995	20170410
12/08	VN075	Northern	22.36618	106.44481	Cao Bang, Duc Xuan commune				20170425
12/09	VN076	Northern	22.36618	106.44481	Cao Bang, Duc Xuan commune				20170426
12/10	VN071	Lang Son	21.71884	106.6127	Lang Son, Mai Sao commune	MG720905	MG720955	MG721003	20170421
12/11	VN045	Lang Son	21.72352	106.60944	Lang Son, Mai Sao commune	MG720902	MG720952	MG721000	20170418
12/12	VN072	Lang Son	21.71884	106.6127	Lang Son, Mai Sao commune	MG720906	MG720956	MG721004	20170422
13/01	H31(2009)a	Ba Be	22.41638	105.63172	Bac Kan, Ba Be National Park	MG720862	MG720912	MG720962	20170404
13/02	B33(2011)c	Ba Be	22.40923	105.62855	Bac Kan, Ba Be National Park	MG720875	MG720925	MG720975	20110515/3
13/03	B8(2011)b	Ba Be	22.44617	105.6174	Bac Kan, Ba Be National Park	MG720872	MG720922	MG720972	20110490/2
13/04	VN094	Ba Be	22.40015	105.62573	Bac Kan, Ba Be National Park				20170427
13/05	VN242	Ba Be	22.40761	105.611	Bac Kan, Ba Be National Park				20170432
13/06	A10(2011)b	Central	21.75523	105.70745	Thai Nguyen, Dong Dat commune	MG720868	MG720918	MG720968	20110481/2
13/07	A10(2011)c	Central	21.75523	105.70745	Thai Nguyen, Dong Dat commune	MG720869	MG720919	MG720969	20110481/3
13/08	A10(2011)d	Central	21.75523	105.70745	Thai Nguyen, Dong Dat commune	MG720870	MG720920	MG720970	20110481/4
13/09	A10(2011)e	Central	21.75523	105.70745	Thai Nguyen, Dong Dat commune				20110481/5
13/10	H2(2009)a	Central	21.7551	105.7073	Thai Nguyen, Dong Dat commune				20170402
14/01	132(2012)a	Southern	17.52843	106.27782	Quang Binh, Phong Nha-Ke Bang National Park	MG720885	MG720935	MG720985	20130927
14/02	157(2012)c	Southern	17.50317	106.26045	Quang Binh, Phong Nha-Ke Bang National Park	MG720886	MG720936	MG720986	20130945/3
14/03	83(2012)b	Southern	17.54147	106.23532	Quang Binh, Phong Nha-Ke Bang National Park				20170406
15/01	VN035	Northern	22.69657	106.37747	Cao Bang, Quoc Dan commune				20170409

* Coordinates based on Schileyko (2011), ** Coordinates estimated based on locality description

Table S2. List of sequence data taken from the literature including taxon and GenBank accession numbers.

Taxon	COI GenBank accession no.	16S GenBank accession no.	28S GenBank accession no.	Reference
Cyclotus sp.	JX474649	JX474739	KF319213	Nantarat et al. (2014b)
Leptopoma vitreum	JX474650	JX474741	KF319214	Nantarat et al. (2014b)
Rhiostoma hainesi	JX474651	JX474740	KF319215	Nantarat et al. (2014b)
Cyclophorus abditus	JX474619	JX474701	KF319183	Nantarat et al. (2014b)
Cyclophorus affinis	JX474590	JX474681	KF319154	Nantarat et al. (2014b)
Cyclophorus amoenus	JX474595	JX474660	KF319159	Nantarat et al. (2014b)
Cyclophorus aurantiacus	JX474642	JX474723	KF319206	Nantarat et al. (2014b)
Cyclophorus bensoni	JX474574	JX474670	KF319138	Nantarat et al. (2014b)
Cyclophorus cantori	JX474629	JX474718	KF319193	Nantarat et al. (2014b)
Cyclophorus cf. *volvulus* (group 1v *sensu* Nantarat et al., 2014b)	JX474586	JX474712	KF319150	Nantarat et al. (2014b)
Cyclophorus cf. *volvulus* (group 2v *sensu* Nantarat et al., 2014b)	JX474609	JX474691	KF319173	Nantarat et al. (2014b)
Cyclophorus cf. *volvulus* (group 3v *sensu* Nantarat et al., 2014b)	JX474602	JX474668	KF319166	Nantarat et al. (2014b)
Cyclophorus consociatus	JX474621	JX474702	KF319185	Nantarat et al. (2014b)
Cyclophorus courbeti	JX474613	JX474695	KF319177	Nantarat et al. (2014b)
Cyclophorus cryptomphalus	JX474594	JX474665	KF319158	Nantarat et al. (2014b)
Cyclophorus diplochilus	JX474624	JX474715	KF319188	Nantarat et al. (2014b)
Cyclophorus expansus	JX474630	JX474719	KF319194	Nantarat et al. (2014b)
Cyclophorus fulguratus	KJ407262	KJ407184	KJ407223	Nantarat et al. (2014c)
Cyclophorus cf. *fulguratus* (group 4f *sensu* Nantarat et al., 2014b)	JX474577	JX474657	KF319141	Nantarat et al. (2014b)
Cyclophorus haughtoni	JX474616	JX474698	KF319180	Nantarat et al. (2014b)
Cyclophorus herklotsi	JX474644	JX474734	KF319208	Nantarat et al. (2014b)
Cyclophorus jourdyi	JX474645	JX474735	KF319209	Nantarat et al. (2014b)
Cyclophorus labiosus	JX474610	JX474692	KF319174	Nantarat et al. (2014b)
Cyclophorus malayanus	JX474571	JX474659	KF319135	Nantarat et al. (2014b)
Cyclophorus perdix tuba	JX474647	JX474737	KF319211	Nantarat et al. (2014b)
Cyclophorus pernobilis	JX474623	JX474722	KF319187	Nantarat et al. (2014b)
Cyclophorus pfeifferi	JX474591	JX474683	KF319155	Nantarat et al. (2014b)
Cyclophorus rangunensis	JX474582	JX474708	KF319146	Nantarat et al. (2014b)
Cyclophorus saturnus	JX474563	JX474677	KF319127	Nantarat et al. (2014b)
Cyclophorus semisulcatus	JX474646	JX474736	KF319210	Nantarat et al. (2014b)
Cyclophorus songmaensis	JX474578	JX474658	KF319142	Nantarat et al. (2014b)
Cyclophorus speciosus	JX474575	JX474655	KF319139	Nantarat et al. (2014b)
Cyclophorus subfloridus	JX474600	JX474666	KF319164	Nantarat et al. (2014b)
Cyclophorus turgidus	JX474643	JX474733	KF319207	Nantarat et al. (2014b)
Cyclophorus zebrinus	JX474632	JX474721	KF319196	Nantarat et al. (2014b)

Table S3. Scores of the first principal component (PC1) from principal component analyses of the morphological dataset and of the following additional morphological datasets: including lineage 15, discarding the size of specimens, including lineage 15 and discarding the size of specimens.

Lineage (Area)	PC1	PC1 (including lineage 15)	PC1 (discarding size)	PC1 (including lineage 15 and discarding size)
01 (Cuc Phuong)	4.98	4.72	-0.05	0.04
02 (Northern)	1.40	1.10	0.02	-0.03
03 (Ba Be)	-2.25	-2.51	-0.01	0.00
03 (Central)	-4.70	-5.02	0.12	-0.13
04 (Lang Son)	0.35	0.01	0.09	-0.10
05 (Coastal)	-4.40	-4.68	0.03	-0.04
06 (Southern)	-6.60	-6.83	-0.03	0.03
07 (Coastal)	-6.23	-6.49	0.06	-0.07
08 (Cuc Phuong)	-1.14	-1.42	0.00	0.00
09 (Central)	-10.25	-10.51	0.10	-0.10
10 (Cuc Phuong)	-4.61	-4.88	0.02	-0.03
11 (Lang Son)	-2.49	-2.75	0.01	-0.01
12 (Coastal)	0.74	0.48	-0.03	0.02
12 (Lang Son)	9.17	8.95	-0.12	0.11
12 (Northern)	10.32	10.01	-0.05	0.04
13 (Ba Be)	4.54	4.28	-0.07	0.06
13 (Central)	3.13	2.84	-0.04	0.02
14 (Southern)	8.04	7.75	-0.05	0.04
15 (Northern)	n.a.	4.93	n.a.	0.16

Table S4. Results of community structure analyses including the standardized effect size (SES) of mean nearest trait distance and respective *p*-values (two-tailed *t*-test) for the following additional morphological datasets: including lineage 15, discarding the size of specimens, including lineage 15 and discarding the size of specimens. Positive SES values indicate overdispersion and negative SES values indicate clustering.

Area	including lineage 15		discarding size		including lineage 15 and discarding size	
	SES (mean nearest trait distance)	*p*-Value for SES of mean nearest trait distance	SES (mean nearest trait distance)	*p*-Value for SES of mean nearest trait distance	SES (mean nearest trait distance)	*p*-Value for SES of mean nearest trait distance
Ba Be	-0.05	.54	-0.24	.46	-0.39	.42
Central	0.94	.18	0.65	.26	0.30	.35
Coastal	-0.76	.26	-0.17	.48	-0.48	.37
Cuc Phuong	-0.03	.51	-0.57	.35	-0.71	.28
Lang Son	0.21	.41	2.17	.03	1.65	.08
Northern	-0.08	.48	-0.14	.52	1.04	.14
Southern	1.66	.09	-1.08	.15	-1.13	.10

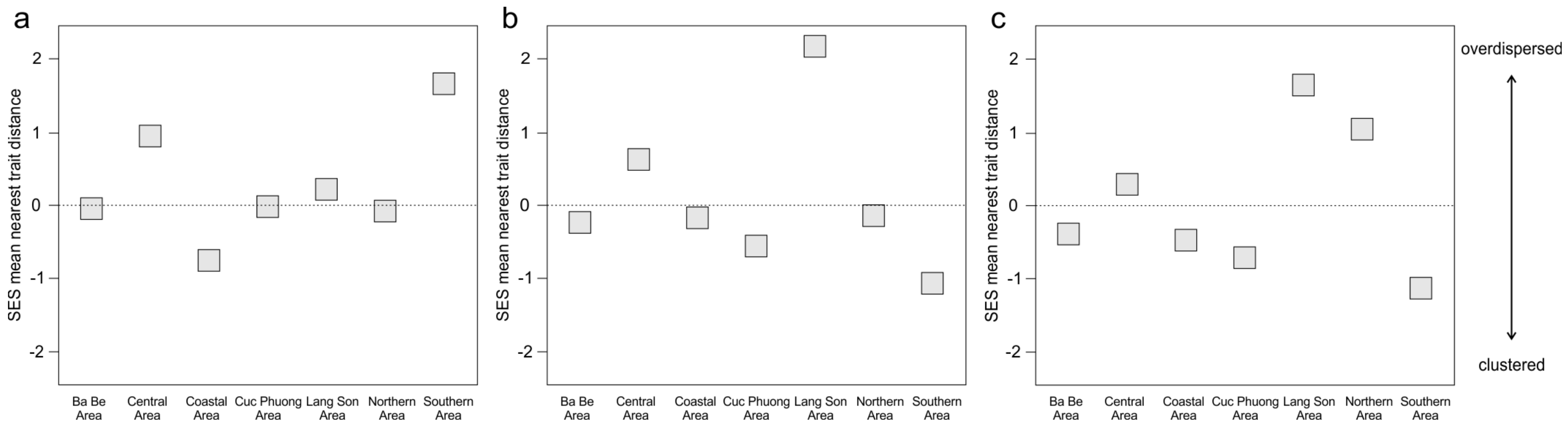

Figure S1. Standardized effect size (SES) of mean nearest trait distance from the following additional morphological datasets: including lineage 15 (a), discarding the size of specimens (b), including lineage 15 and discarding the size of specimens (c). Positive SES values indicate overdispersion and negative SES values indicate clustering.
